# Quercetin attenuates polystyrene nanoplastic-induced intestinal barrier injury through modulation of macrophage inflammatory responses

**DOI:** 10.3389/fphar.2026.1973458

**Published:** 2026-10-12

**Authors:** Jian Kang, Heng Yang, Yan Wang, Xin Zhang, Chunyang Zhu, Yanhong Zhou, Yanqin Li

**Affiliations:** 1 Guangdong Polytechnic of Science and Trade, Guangzhou, Guangdong, China; 2 National Key Laboratory of Agricultural Microbiology, Huazhong Agricultural University, Wuhan, Hubei, China; 3 Guangzhou Zoo and Guangzhou Wildlife Research Center, Guangzhou, Guangdong, China; 4 Guangdong Eco-engineering Polytechnic, Guangzhou, Guangdong, China

**Keywords:** macrophage polarization, NF-κB, polystyrene nanoplastics, quercetin, STAT1

## Abstract

**Introduction:**

Polystyrene nanoplastics (PS-NPs) are emerging environmental contaminants that may impair intestinal barrier integrity, but the involvement of macrophage inflammatory responses remains incompletely understood. Quercetin is a natural flavonoid with well-established anti-inflammatory and immunomodulatory properties. This study investigated the role of macrophage inflammatory responses in PS-NP-induced intestinal injury and evaluated the protective effects of quercetin.

**Methods:**

Male ICR mice were orally exposed to PS-NPs for 4 consecutive weeks, and the effects of quercetin intervention on colonic injury, inflammatory responses, tight junction-associated proteins, and macrophage polarization markers were evaluated. RAW 264.7 macrophages were used to further examine PS-NP-induced inflammatory responses and the effects of quercetin *in vitro*. An indirect Transwell co-culture system of RAW 264.7 macrophages and MODE-K intestinal epithelial cells was established to assess macrophage-mediated epithelial barrier alterations. NF-κB and STAT1 signaling activation was also examined.

**Results:**

PS-NP exposure induced dose-dependent colonic histopathological injury, increased the expression of the pro-inflammatory cytokines IL-1β, TNF-α, and IL-6, and reduced the expression of the tight junction proteins ZO-1 and occludin. PS-NPs were also associated with a shift toward a pro-inflammatory macrophage phenotype, characterized by increased CD86 and decreased CD206 expression. Consistently, PS-NPs promoted inflammatory responses in RAW 264.7 macrophages and increased NF-κB p65 and STAT1 phosphorylation. In the Transwell co-culture system, MODE-K cells co-cultured with PS-NP-stimulated macrophages exhibited reduced tight junction-associated protein expression and disrupted junctional integrity. Quercetin treatment attenuated PS-NP-induced colonic injury and inflammatory responses, partially restored ZO-1 and occludin expression, modulated macrophage polarization-associated markers, and reduced NF-κB and STAT1 activation.

**Discussion:**

These findings indicate that 50 nm PS-NP exposure induces intestinal inflammatory responses and barrier-associated injury accompanied by pro-inflammatory macrophage activation and altered NF-κB/STAT1 signaling. The co-culture findings further support the involvement of macrophage-derived inflammatory responses in epithelial barrier impairment. Quercetin attenuates PS-NP-induced intestinal injury, potentially in part through modulation of macrophage inflammatory responses accompanied by reduced NF-κB and STAT1 activation.

## 1 Introduction

The intestinal barrier constitutes a critical defense against the entry of harmful exogenous substances (Turner, 2009). It is formed by intestinal epithelial cells, tight junction-associated proteins, including occludin and zonula occludens-1 (ZO-1), and mucosal immune cells, whose coordinated structural and functional integrity is indispensable for maintaining intestinal homeostasis (Odenwald and Turner, 2017; Chelakkot et al., 2018). In recent years, nanoplastics have emerged as environmental contaminants of increasing concern because of their small size, large specific surface area, high tissue penetration potential, and propensity for biological accumulation. Although a universally accepted definition has yet to be established, nanoplastics are generally considered plastic particles smaller than 1 μm, while an upper size limit of 100 nm is also adopted in some studies (Gigault et al., 2018; Lai et al., 2022). Polystyrene nanoplastics (PS-NPs), one of the most widely studied types of nanoplastics, can enter the body through the gastrointestinal tract and interact directly with the intestinal mucosa (Liang et al., 2021). PS-NPs exposure has been shown to induce local inflammation, intestinal epithelial injury, tight junction disruption, and increased intestinal permeability, ultimately leading to intestinal barrier dysfunction (He et al., 2022). However, the immunoregulatory mechanisms underlying PS-NP-induced intestinal barrier injury remain unclear.

Macrophages constitute a major immune cell population in the intestinal lamina propria and exhibit considerable phenotypic plasticity (Mowat and Bain, 2011). In response to inflammatory stimuli, macrophages can acquire a pro-inflammatory M1-like phenotype characterized by increased expression of CD86 and iNOS, together with enhanced secretion of TNF-α, IL-1β, and IL-6 (Murray et al., 2014; Jiang et al., 2024). These pro-inflammatory mediators can disrupt tight junction regulation, increase epithelial permeability, and thereby aggravate intestinal barrier injury (Al-Sadi et al., 2010; Al-Sadi et al., 2013; Al-Sadi et al., 2014). Growing evidence suggests that pro-inflammatory macrophage polarization is an important contributor to pollutant-induced inflammatory injury, and PS-NPs have been shown to promote M1-like macrophage polarization in experimental models (Li et al., 2023). However, whether macrophage inflammatory polarization contributes to PS-NPs-induced intestinal barrier injury and the extent of its involvement remain unclear.

Quercetin is a naturally occurring flavonoid widely distributed in fruits and vegetables and exhibits potent antiinflammatory, antioxidant, and immunomodulatory activities (Cho et al., 2003). Previous studies have shown that quercetin exhibits anti-inflammatory and immunomodulatory activities and may regulate macrophage inflammatory responses through pathways including NF-κB- and STAT1-associated signaling (Tsai et al., 2021; Gao et al., 2024; Wang et al., 2024). However, whether its protective effect against PS-NPs induced intestinal barrier injury is specifically mediated by the suppression of macrophage M1 polarization remains unclear. Accordingly, this study aimed to investigate PS-NP-induced intestinal barrier injury and the involvement of macrophage inflammatory responses, and to evaluate whether quercetin could alleviate intestinal injury by modulating inflammatory processes. These findings may provide a theoretical basis for preventing nanoplastic-induced intestinal toxicity and developing natural product-based intervention strategies.

## 2 Materials and methods

### 2.1 Animals and experimental design

All animal experimental protocols in this study were approved by the Animal Ethics Committee of Guangdong Polytechnic of Science and Trade. Male ICR mice (4 weeks old) were purchased from Beijing Vital River Laboratory Animal Technology Co., Ltd (Beijing, China). Male mice were selected to maintain comparability with previous experimental studies investigating PS-NP-induced intestinal effects and quercetin intervention in male mice, and to focus the present mechanistic investigation on a single sex (Zhao et al., 2024). The mice were housed in individually ventilated cages under controlled conditions of 23 °C ± 1 °C, 55% ± 5% relative humidity, and a 12 h light/12 h dark cycle, with *ad libitum* access to food and water. After 1 week of acclimatization, the mice were used for subsequent experiments. Polystyrene nanoparticles (PS-NPs, nominal diameter: 50 nm) were purchased from Beijing Biotyscience Co., Ltd (Beijing, China; catalog No. ABT-8–0005). According to the manufacturer, the stock suspension had a concentration of 5.0%. Before administration, PS-NPs were dispersed by ultrasonication to obtain a homogeneous suspension. Quercetin (purity ≥98%) was dissolved in corn oil and freshly prepared before use.

For the dose-selection experiment, the mice were randomly assigned to three groups (*n* = 6 per group): the control group (0 mg/kg/day), the low-dose PS-NP group (0.5 mg/kg/day), and the high-dose PS-NP group (5 mg/kg/day). The PS-NP exposure doses were selected with reference to previous experimental studies of PS-NP-induced intestinal toxicity and were further evaluated through the dose-response experiment described above. These doses were used as experimental exposure levels for investigating biological effects rather than as direct representations of environmentally relevant exposure. The mice received the corresponding doses of PS-NPs once daily by oral gavage for four consecutive weeks. For the intervention experiment, the mice were randomly divided into four groups (*n* = 6 per group; The animal sample size was determined based on previous studies using comparable mouse models of nanoplastic-induced intestinal injury, preliminary experimental observations, and ethical considerations aimed at minimizing animal use while obtaining sufficient biological information.): the control group, quercetin group, PS-NPs group, and PS-NPs + quercetin group. Mice in the PS-NPs and PS-NPs + quercetin groups were administered PS-NPs at 5 mg/kg/day by oral gavage for 4 weeks. Beginning in the third week, mice in the quercetin and PS-NPs + quercetin groups received quercetin at 200 mg/kg/day by oral gavage for two consecutive weeks. The quercetin dose was selected as an experimental intervention dose with reference to previous rodent studies in which relatively high oral doses of quercetin were used to produce measurable biological effects (Wu et al., 2017) Pharmacokinetic studies in rats have further demonstrated that oral quercetin at relatively high doses produces measurable circulating quercetin and conjugated metabolites, despite its extensive metabolism and limited systemic availability (Moon et al., 2001; Yang et al., 2016). Groups not receiving PS-NPs or quercetin were administered equivalent volumes of the corresponding vehicles (water for PS-NP suspension and corn oil for quercetin administration), and the overall experimental duration was identical among groups and the overall experimental duration was identical among groups. At the end of the experiment, the mice were fasted for 12 h with free access to water and sacrificed by cervical dislocation. Blood samples were collected, and serum was isolated for subsequent analyses. Colon tissues were harvested, with one portion fixed in 4% paraformaldehyde for histological examination and the remaining tissue immediately snap-frozen in liquid nitrogen and stored at −80 °C for subsequent gene and protein expression analyses.

### 2.2 Cell culture and treatments

Murine macrophage RAW 264.7 cells and murine small intestinal epithelial MODE-K cells were cultured in high-glucose Dulbecco’s modified Eagle’s medium supplemented with 10% fetal bovine serum, 100 U/mL penicillin, and 100 μg/mL streptomycin. Cells were maintained at 37 °C in a humidified atmosphere containing 5% CO_2_.

To determine an appropriate exposure concentration, RAW 264.7 cells were treated with PS-NPs at 0, 10, 50, or 100 μg/mL for 24 h. For the intervention experiments, RAW 264.7 cells were pretreated with quercetin at 5, 10, or 20 μM for 2 h, followed by exposure to 100 μg/mL PS-NPs in the continued presence of quercetin for 24 h. The concentration range used for the macrophage experiments was selected with reference to previous *in vitro* studies investigating PS-NP-induced inflammatory responses in RAW 264.7 macrophages (Chen et al., 2023), together with the concentration-response results obtained in the present study.

To evaluate the potential cytotoxic effects of PS-NPs and quercetin under the experimental conditions used in this study, RAW 264.7 macrophages and MODE-K intestinal epithelial cells were treated with PS-NPs (100 μg/mL) or quercetin (20 μM) for 24 h. Cell viability was assessed using the Cell Counting Kit-8 (CCK-8) assay according to the manufacturer’s instructions. Briefly, CCK-8 reagent was added after treatment, and absorbance was measured at 450 nm using a microplate reader. Cell viability was calculated as a percentage relative to the control group.

A Transwell co-culture system was established to investigate macrophage-mediated epithelial injury. RAW 264.7 cells treated with 100 μg/mL PS-NPs alone or in combination with 20 μM quercetin for 24 h were seeded into the upper inserts, while MODE-K cells were seeded into the lower chambers. A Transwell insert with a 0.4 μm pore-size membrane was used to establish an indirect co-culture system, allowing the exchange of macrophage-derived soluble factors while preventing direct physical contact between RAW 264.7 macrophages and MODE-K intestinal epithelial cells. The 2 cell types were subsequently co-cultured for an additional 24 h.

### 2.3 Histopathological examination

Colon tissues were fixed in 4% paraformaldehyde for 24 h, routinely dehydrated, cleared, embedded in paraffin, and cut into serial 5 μm sections. The sections were stained with H and E according to standard procedures. Colonic morphology, epithelial integrity, and inflammatory cell infiltration were examined under a light microscope.

### 2.4 qRT-PCR

Total RNA was extracted from cells and colon tissues using TRIzol reagent, and complementary DNA was synthesized using a reverse transcription kit. The relative mRNA expression levels of *TNF-α, IL-1β*, and *IL-6* were determined by real-time quantitative PCR, with *Actb* serving as the internal reference gene. Relative gene expression was calculated using the 2^−ΔΔCt^ method. The primers used in the experiment are listed in Table 1.

**TABLE 1:** Primers used for quantitative RT-PCR.

Genes	Primer sequences (5′–3′)	Product length (bp)	Accession no.
*IL-1β*	F: GCAACTGTTCCTGAACTCAACT R: ATCTTTTGGGGTCCGTCAACT	89	NM_008361.4
*TNF-α*	F: CAGGCGGTGCCTATGTCTC R: CGATCACCCCGAAGTTCAGTAG	89	NM_013693.4
*IL-6*	F: TAGTCCTTCCTACCCCAATTTCC R: TTGGTCCTTAGCCACTCCTTC	76	NM_031168.2
*β-actin*	F: GGCTGTATTCCCCTCCATCG R: CCAGTTGGTAACAATGCCATGT	154	NM_007393.5

### 2.5 Western blotting

Total proteins were extracted from cells and colon tissues using RIPA lysis buffer supplemented with phenylmethylsulfonyl fluoride, and protein concentrations were determined using a bicinchoninic acid assay kit. Equal amounts of protein were separated by sodium dodecyl sulfate–polyacrylamide gel electrophoresis and transferred onto polyvinylidene fluoride membranes. After blocking with 5% non-fat milk for 2 h at room temperature, the membranes were incubated overnight at 4 °C with primary antibodies against ZO-1 (21773-1-AP), Occludin (27260-1-AP), CD86 (27306-1-AP), NF-κB p65 (10745-1-AP), phosphorylated NF-κB p65 (p-NF-κB p65; 30336), STAT1 (10144-2-AP), phosphorylated STAT1 (p-STAT1; 28977-1-AP), and β-actin (66009-1-Ig). After washing with Tris-buffered saline containing Tween 20, the membranes were incubated with the corresponding secondary antibodies for 1 h at room temperature. Protein bands were visualized using an enhanced chemiluminescence detection system, and band intensities were quantified using ImageJ software.

### 2.6 Immunofluorescence staining

Following co-culture, MODE-K cells were fixed with 4% paraformaldehyde, permeabilized with 0.1% Triton X-100, and blocked with 5% bovine serum albumin. The cells were incubated overnight at 4 °C with an anti-occludin primary antibody (1:100; 27260-1-AP), followed by incubation with an appropriate fluorescence-conjugated secondary antibody in the dark. Nuclei were counterstained with DAPI, and the samples were mounted for imaging. The expression and subcellular distribution of occludin were examined using a fluorescence microscope.

### 2.7 Statistical analysis

For the animal experiments, each mouse was considered an independent biological replicate (*n* = 6 per group). *In vitro* experiments were independently repeated at least three times, with each independent experiment considered a biological replicate. Technical replicates within each independent experiment were averaged before statistical analysis. Data are presented as the mean ± standard deviation. Statistical analyses were performed using IBM SPSS Statistics version 29.0. Comparisons between two groups were conducted using an independent-samples Student’s t-test. Comparisons among multiple groups were performed using one-way or two-way analysis of variance according to the experimental design, followed by Tukey’s multiple comparisons test when appropriate. For experiments involving two independent factors, two-way ANOVA was applied to evaluate the main effects of each factor and their interaction. A *P*-value <0.05 was considered statistically significant.

## 3 Result

### 3.1 Effects of PS-NPs exposure on intestinal barrier integrity and inflammatory responses in mice

To evaluate the intestinal effects of PS-NPs exposure at different doses, mice were assigned to a control group, a low-dose PS-NPs group (0.5 mg/kg/day), and a high-dose PS-NPs group (5 mg/kg/day) and were exposed by oral gavage for four consecutive weeks. As shown in Figure 1A, the colonic tissue of control mice exhibited an intact mucosal architecture, regularly arranged epithelial cells, and well-defined crypt structures. PS-NPs exposure resulted in progressively increased histopathological alterations with increasing dose. The low-dose group showed mild mucosal edema and increased inflammatory cell infiltration, whereas the high-dose group exhibited epithelial shedding, crypt disruption, and marked architectural disorganization, indicating a dose-dependent aggravation of colonic histopathological injury.

**FIGURE 1:**
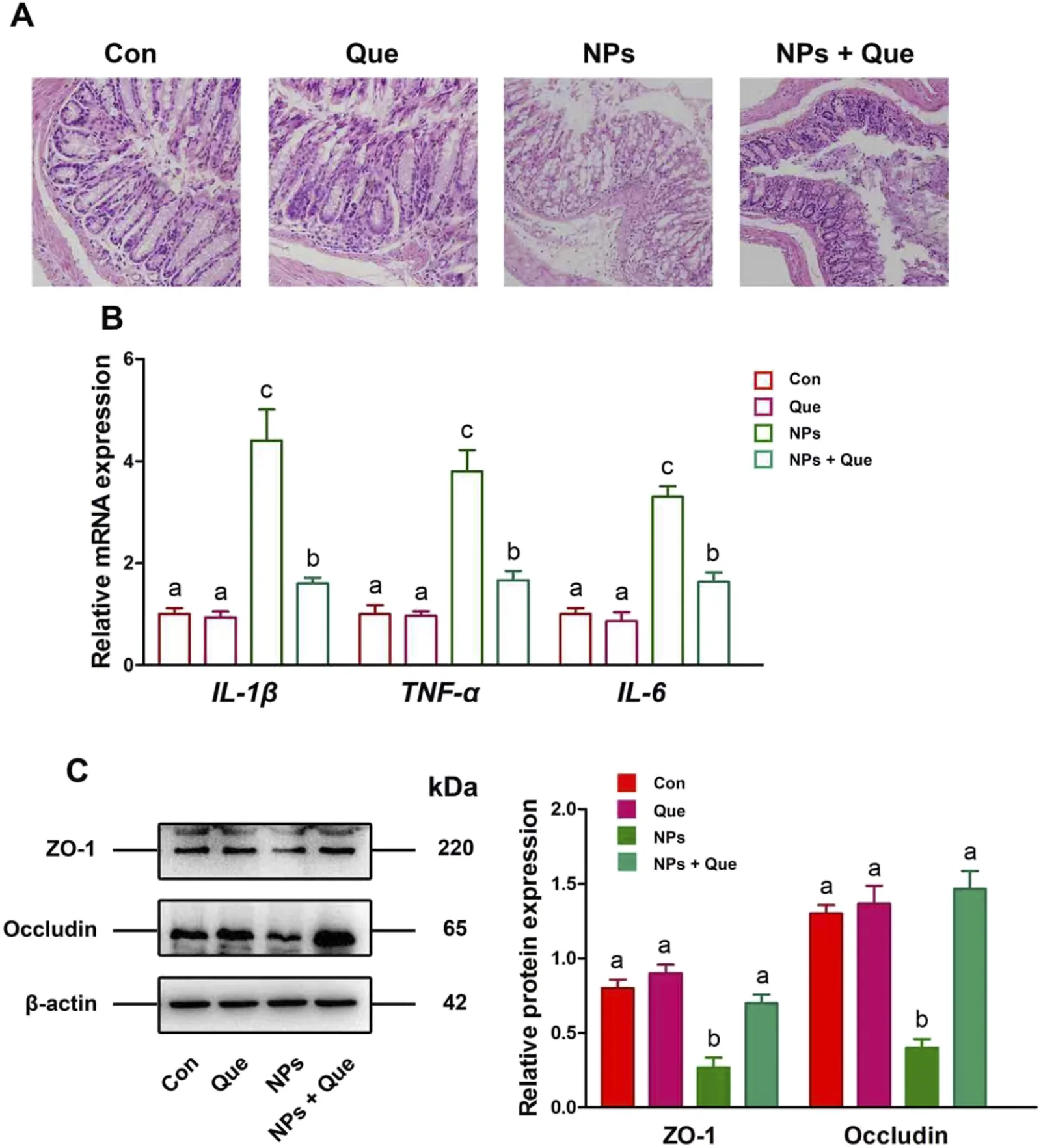
Effects of PS-NP exposure on colonic histology, inflammatory cytokine expression, and tight junction protein levels in mice. Mice were orally administered PS-NPs at 0, 0.5, or 5 mg/kg/day for four consecutive weeks. **(A)** Histopathological changes in the colon of mice exposed to different doses of PS-NPs. **(B)** Changes in the mRNA expression levels of the pro-inflammatory cytokines *TNF-α, IL-1β*, and *IL-6* in the colon following exposure to different doses of PS-NPs. **(C)** Changes in the protein expression levels of the tight junction proteins ZO-1 and occludin in the colon following exposure to different doses of PS-NPs. β-actin was used as the loading control. Values are presented as mean ± SD (*n* = 6 per group). Different letters indicate significant differences among groups (*P* < 0.05).

The mRNA expression levels of intestinal inflammatory mediators were subsequently examined. As shown in Figure 1B, PS-NPs exposure significantly increased the expression of *TNF-α, IL-1β*, and *IL-6* compared with the control group, with greater increases observed at the higher dose (*P* < 0.05). These findings indicated that PS-NPs exposure elicited a local intestinal inflammatory response. Western blot analysis further showed that the expression levels of the tight junction-associated proteins ZO-1 and occludin were significantly reduced following PS-NPs exposure in a dose-dependent manner (Figure 1C). Collectively, these results indicated that PS-NPs exposure was associated with intestinal inflammation and disruption of tight junction-associated proteins, suggesting impaired colonic barrier integrity. Because the 5 mg/kg/day PS-NPs exposure produced more pronounced histopathological alterations and inflammatory responses, this dose was selected for subsequent intervention experiments.

### 3.2 Protective effects of quercetin against PS-NP-induced intestinal injury in mice

Based on the dose-selection results, 5 mg/kg/day PS-NPs was selected to induce intestinal injury and evaluate the protective effects of quercetin. As shown in Figure 2A, the colonic architecture remained intact in the control and quercetin groups, with regularly arranged mucosal epithelial cells. In contrast, mice in the PS-NPs group exhibited marked histopathological injury, including mucosal edema, epithelial shedding, and crypt disorganization. Quercetin treatment markedly attenuated these alterations, as evidenced by improved mucosal architecture and reduced inflammatory cell infiltration, suggesting that quercetin alleviated PS-NPs induced colonic injury.

**FIGURE 2:**
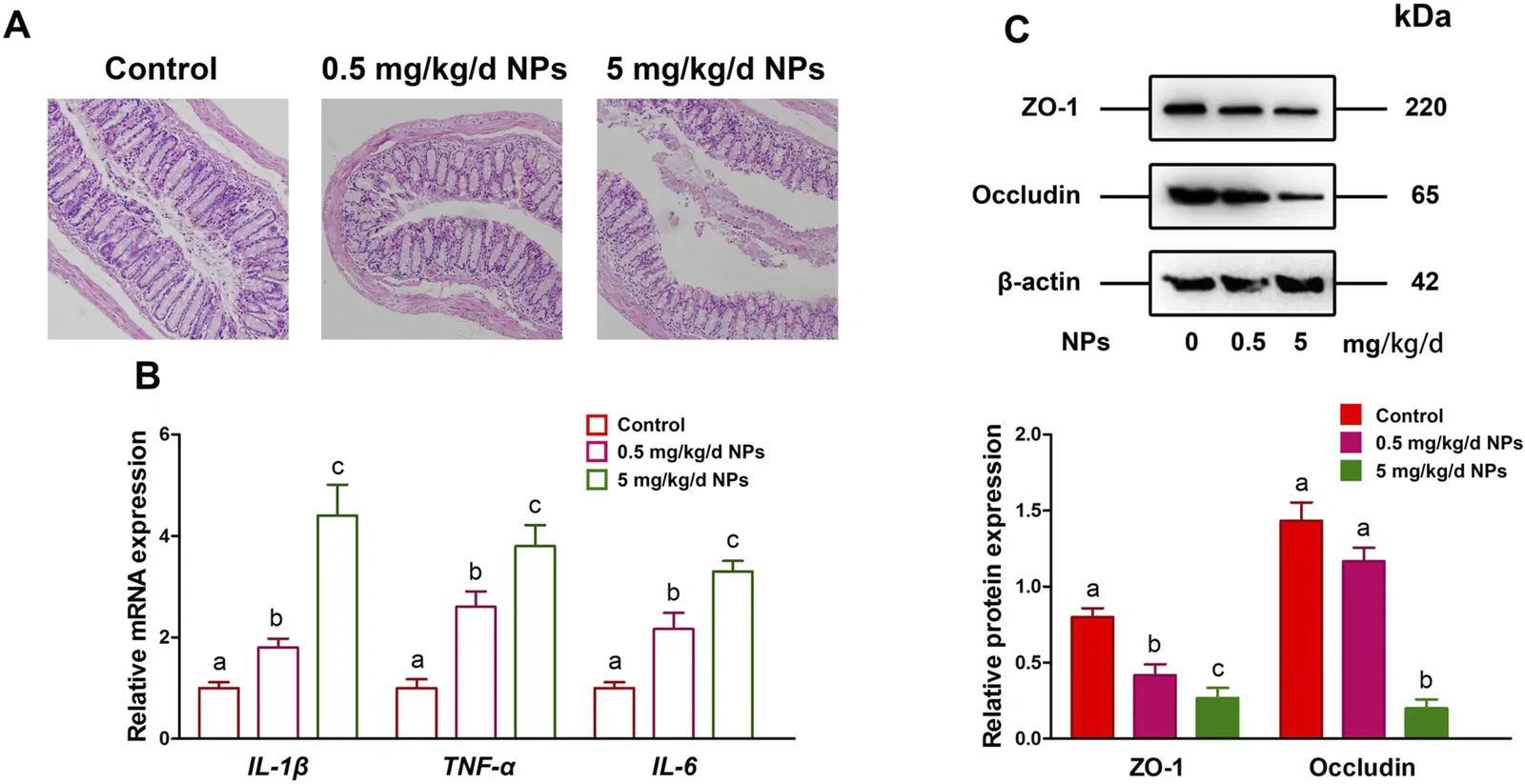
Protective effects of quercetin against PS-NP-induced colonic injury in mice. **(A)** Colonic histopathological changes in control mice and mice treated with quercetin, PS-NPs, or PS-NPs combined with quercetin. **(B)** Changes in the colonic mRNA expression levels of the pro-inflammatory cytokines *TNF-α, IL-1β*, and *IL-6* following quercetin intervention in PS-NP-exposed mice. **(C)** Changes in the colonic protein expression levels of ZO-1 and occludin following quercetin intervention in PS-NP-exposed mice. β-actin was used as the loading control. Values are presented as mean ± SD (*n* = 6 per group). Different letters indicate significant differences among groups (*P* < 0.05).

As shown in Figure 2B, the mRNA expression levels of *TNF-α, IL-1β*, and *IL-6* were significantly increased in the PS-NPs group compared with the control group (*P* < 0.05). Quercetin treatment significantly reduced the expression of these pro-inflammatory mediators relative to the PS-NPs group (*P* < 0.05), whereas no significant differences were observed between the quercetin and control groups. These results indicated that quercetin attenuated PS-NP-induced intestinal inflammatory responses. Western blot analysis further showed that PS-NPs exposure significantly decreased the protein expression levels of ZO-1 and occludin in colonic tissues, whereas quercetin treatment significantly partially recovered their expression (Figure 2C). Collectively, these findings suggested that quercetin attenuated PS-NP-induced colonic injury, reduced intestinal inflammatory responses, and preserved tight junction-associated protein expression, indicating a protective effect against intestinal barrier impairment.

### 3.3 Effects of quercetin on PS-NP-induced macrophage inflammatory polarization in the mouse intestine

To investigate whether macrophage polarization was associated with the intestinal protective effects of quercetin, the protein expression levels of the M1-associated marker CD86 and the M2-associated marker CD206 were examined in colonic tissues. Compared with the control group, the PS-NPs group showed significantly increased CD86 expression and decreased CD206 expression (*P* < 0.05), suggesting that PS-NPs exposure was associated with a shift toward a pro-inflammatory macrophage phenotype in colonic tissues. Quercetin treatment significantly attenuated these changes, as demonstrated by reduced CD86 expression and increased CD206 expression in the PS-NPs + quercetin group compared with the PS-NPs group (*P* < 0.05). No significant differences were observed between the quercetin and control groups (Figure 3). These findings suggested that quercetin attenuated the PS-NP-associated pro-inflammatory macrophage response and modulated the expression profile of M1-and M2-associated markers, which may contribute to its protective effects against intestinal inflammation and barrier impairment.

**FIGURE 3:**
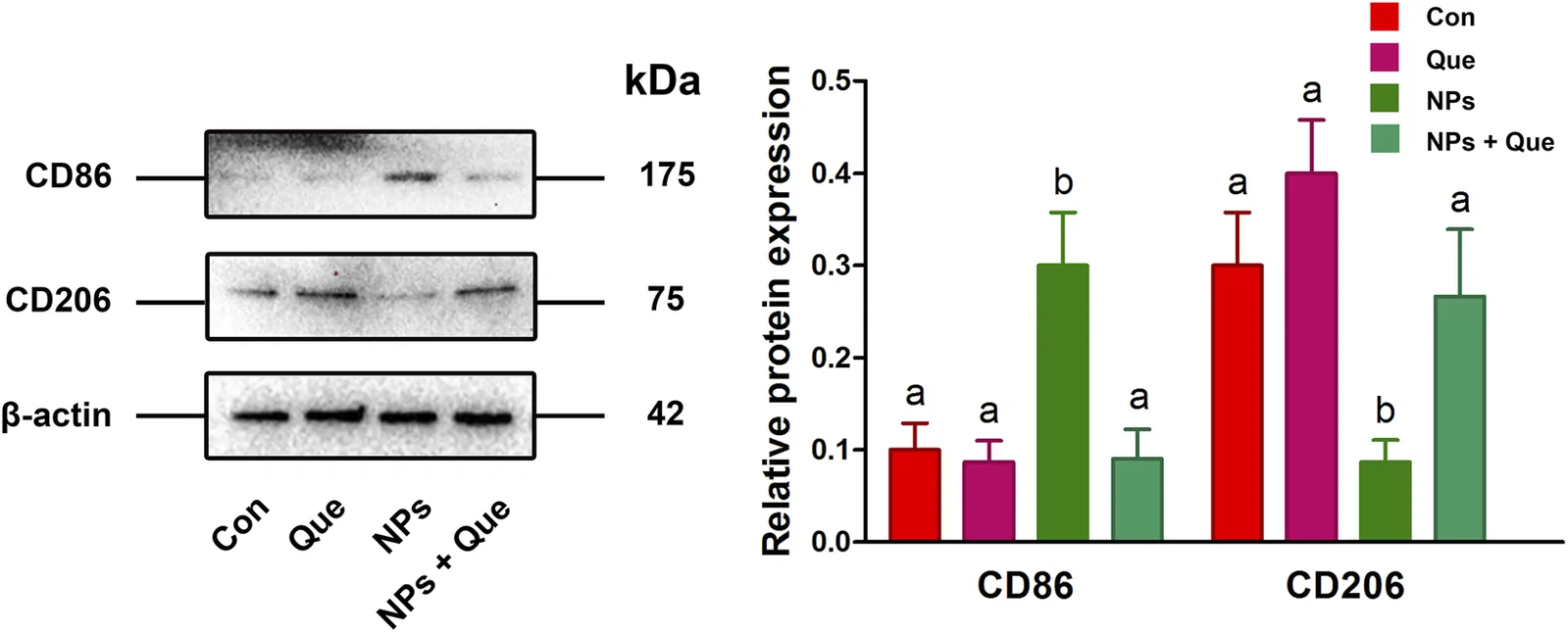
Effects of quercetin on PS-NP-induced changes in macrophage polarization markers in mouse colon. Changes in the protein expression levels of the M1-associated marker CD86 and the M2-associated marker CD206 in the colonic tissues of control mice and mice treated with quercetin, PS-NPs, or PS-NPs combined with quercetin. β-actin was used as the loading control. Values are presented as mean ± SD (*n* = 6 per group). Different letters indicate significant differences among groups (*P* < 0.05).

### 3.4 PS-NPs promote a pro-inflammatory macrophage phenotype in RAW 264.7 cells

To investigate whether PS-NPs exposure affects macrophage inflammatory responses, RAW 264.7 cells were exposed to PS-NPs at 0, 10, 50, or 100 μg/mL for 24 h. As shown in Figure 4A, treatment with 50 and 100 μg/mL PS-NPs significantly increased the mRNA expression levels of *TNF-α, IL-1β*, and *IL-6* compared with the control group, with greater increases observed at higher concentrations, indicating enhanced inflammatory activation of RAW 264.7 macrophages. Western blot analysis further showed that PS-NPs exposure increased the protein expression of the M1-associated marker CD86 while decreasing that of the M2-associated marker CD206 (Figure 4B), suggesting that PS-NPs exposure was associated with a shift toward a pro-inflammatory macrophage phenotype in RAW 264.7 cells. Because 100 μg/mL PS-NPs produced the most pronounced inflammatory response, this concentration was selected for subsequent intervention experiments.

**FIGURE 4:**
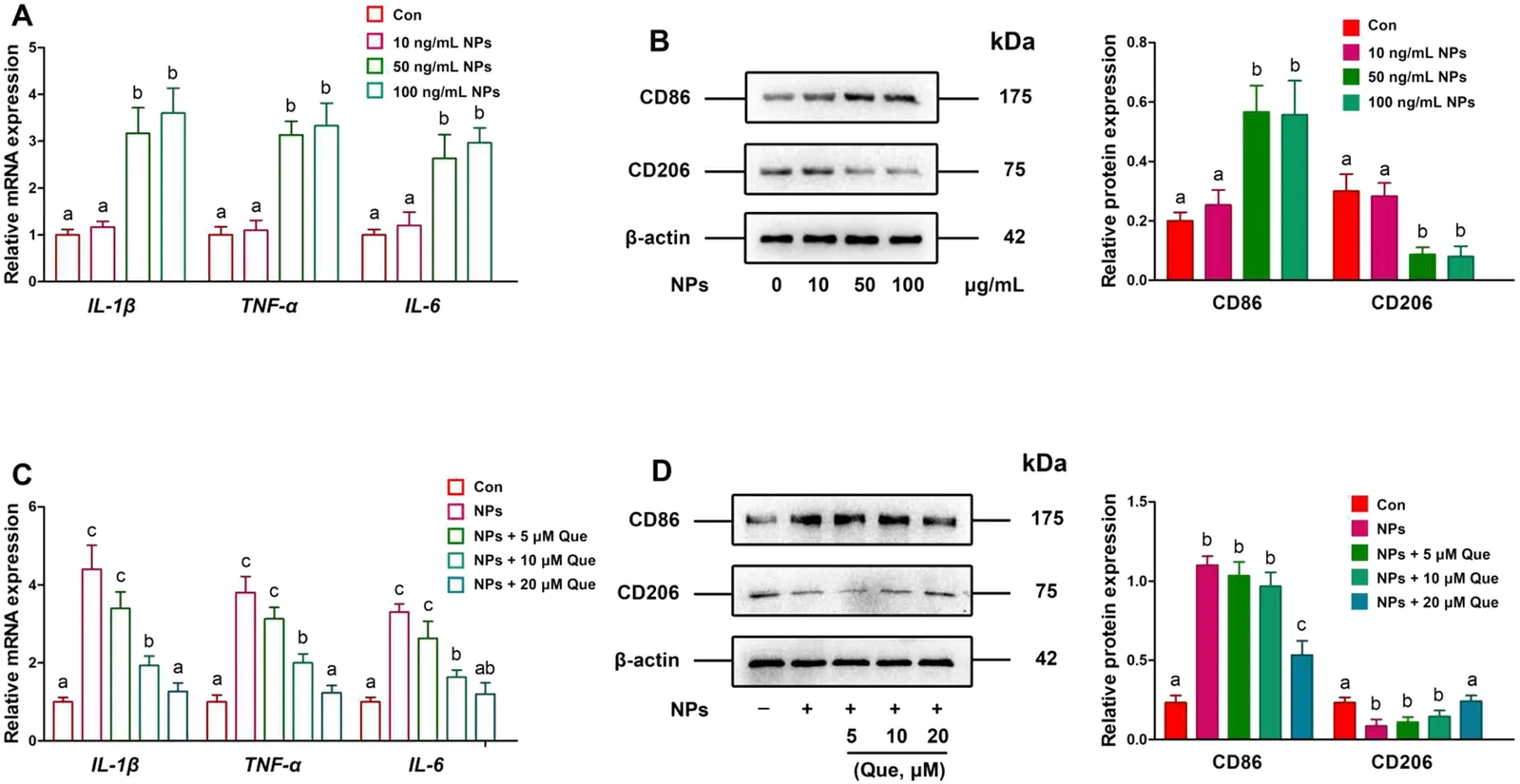
Effects of PS-NPs and quercetin on inflammatory responses and polarization-related markers in RAW 264.7 macrophages. **(A)** Changes in the mRNA expression levels of *TNF-α, IL-1β*, and *IL-6* in RAW 264.7 macrophages treated with different concentrations of PS-NPs. **(B)** Changes in the protein expression levels of the M1-associated marker CD86 and the M2-associated marker CD206 following treatment with different concentrations of PS-NPs. **(C)** Changes in the mRNA expression levels of *TNF-α, IL-1β*, and *IL-6* in PS-NP-exposed RAW 264.7 macrophages treated with different concentrations of quercetin **(D)** Changes in CD86 and CD206 protein expression in PS-NP-exposed RAW 264.7 macrophages treated with different concentrations of quercetin. β-actin was used as the loading control. Values are presented as mean ± SD from three independent experiments (*n* = 3). Different letters indicate significant differences among groups (*P* < 0.05).

The intervention effects of quercetin were further investigated. As shown in Figures 4C,D, quercetin dose-dependently reduced the PS-NPs induced increases in *TNF-α, IL-1β*, and *IL-6* mRNA expression. Quercetin treatment also decreased CD86 protein expression and increased CD206 protein expression compared with PS-NPs exposure alone, suggesting attenuation of the PS-NP-associated pro-inflammatory macrophage phenotype. Collectively, these findings indicated that PS-NPs directly promoted a pro-inflammatory macrophage response in RAW 264.7 cells, whereas quercetin attenuated this response and modulated the expression of M1-and M2-associated markers.

### 3.5 Quercetin attenuates PS-NP-induced intestinal epithelial barrier injury through modulation of macrophage inflammatory responses

To further investigate whether macrophage inflammatory responses contribute to intestinal epithelial barrier impairment, a Transwell co-culture system was established using RAW 264.7 macrophages and MODE-K intestinal epithelial cells. RAW 264.7 cells treated with PS-NPs alone or in combination with quercetin were co-cultured with MODE-K cells to evaluate the indirect effects of macrophage inflammatory responses on epithelial barrier-associated changes. Western blot analysis showed that, compared with MODE-K cells cultured alone, MODE-K cells co-cultured with PS-NPs treated RAW 264.7 macrophages exhibited significantly reduced expression of the tight junction proteins ZO-1 and occludin (Figure 5A). These findings suggested that soluble factors released from PS-NP-stimulated macrophages contributed to disruption of epithelial tight junction-associated proteins in MODE-K cells under an indirect co-culture condition. In contrast, co-culture with quercetin-treated macrophages significantly restored ZO-1 and occludin expression in MODE-K cells, suggesting that quercetin attenuated macrophage-mediated epithelial barrier impairment through modulation of the PS-NPs-associated pro-inflammatory macrophage phenotype. Immunofluorescence staining further supported these findings (Figure 5B). In control MODE-K cells, ZO-1 exhibited a continuous and uniform distribution along the cell membrane. Following co-culture with PS-NP treated macrophages, ZO-1 fluorescence intensity was markedly reduced and its membrane distribution became discontinuous. By contrast, both ZO-1 fluorescence intensity and junctional continuity were substantially restored in MODE-K cells co-cultured with quercetin-treated macrophages, consistent with the Western blot results. Collectively, these findings support the involvement of PS-NP-associated macrophage inflammatory responses in intestinal epithelial barrier impairment and indicate that quercetin protects epithelial barrier-associated features, at least in part, through modulation of macrophage inflammatory responses.

**FIGURE 5:**
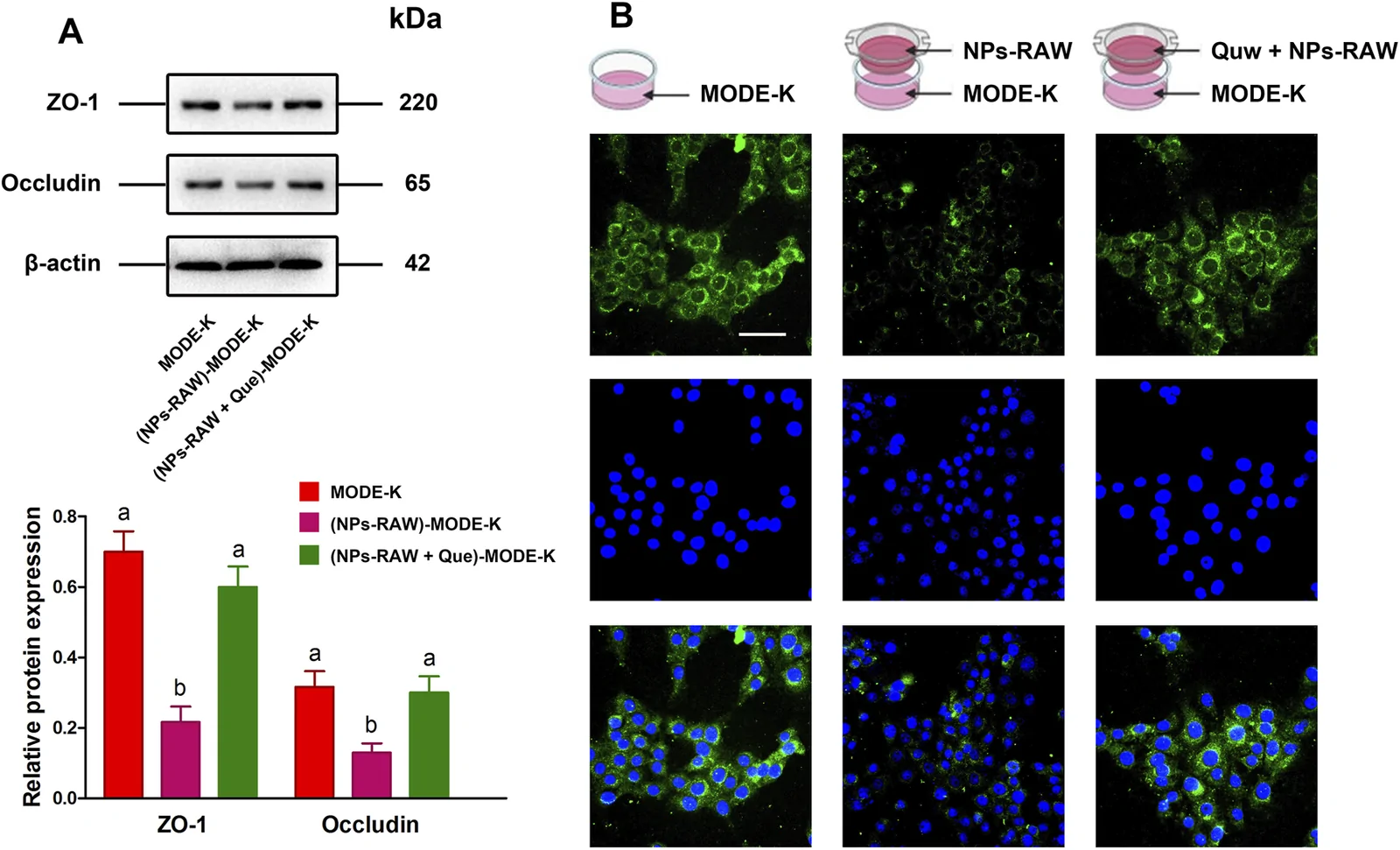
Effects of quercetin-treated macrophages on tight junction integrity in MODE-K intestinal epithelial cells under PS-NP exposure conditions. **(A)** Changes in ZO-1 and occludin protein expression in MODE-K cells cultured alone or co-cultured with PS-NP-treated RAW 264.7 macrophages in the absence or presence of quercetin. β-actin was used as the loading control. **(B)** Changes in occludin localization and fluorescence intensity in MODE-K cells under the corresponding co-culture conditions. Occludin is shown in green, and nuclei were counterstained with DAPI (blue). Original magnification: ×200; scale bar = 100 μm. Values are presented as mean ± SD from three independent experiments (*n* = 3). Different letters indicate significant differences among groups (*P* < 0.05).

### 3.6 Quercetin modulates macrophage inflammatory responses accompanied by altered NF-κB and STAT1 activation

To investigate the signaling events associated with quercetin-mediated modulation of PS-NP-induced macrophage inflammatory responses, the phosphorylation levels of key proteins in the NF-κB and STAT1 signaling pathways were examined in RAW 264.7 cells. Western blot analysis (Figure 6) showed that PS-NPs treatment significantly increased the phosphorylation levels of NF-κB p65 and STAT1 compared with the control group, as reflected by elevated p-NF-κB p65/NF-κB p65 and p-STAT1/STAT1 ratios (*P* < 0.05). These findings indicated that PS-NPs exposure was associated with increased NF-κB and STAT1 activation, which may contribute to the induction of a pro-inflammatory macrophage phenotype. Quercetin treatment reduced the phosphorylation levels of NF-κB p65 and STAT1 compared with PS-NPs exposure alone, indicating altered activation status of these inflammatory signaling pathways. Collectively, these results suggested that quercetin attenuated PS-NP-induced macrophage inflammatory responses, accompanied by reduced NF-κB and STAT1 activation. These signaling changes may contribute to the anti-inflammatory effects of quercetin and its protective effects against intestinal injury.

**FIGURE 6:**
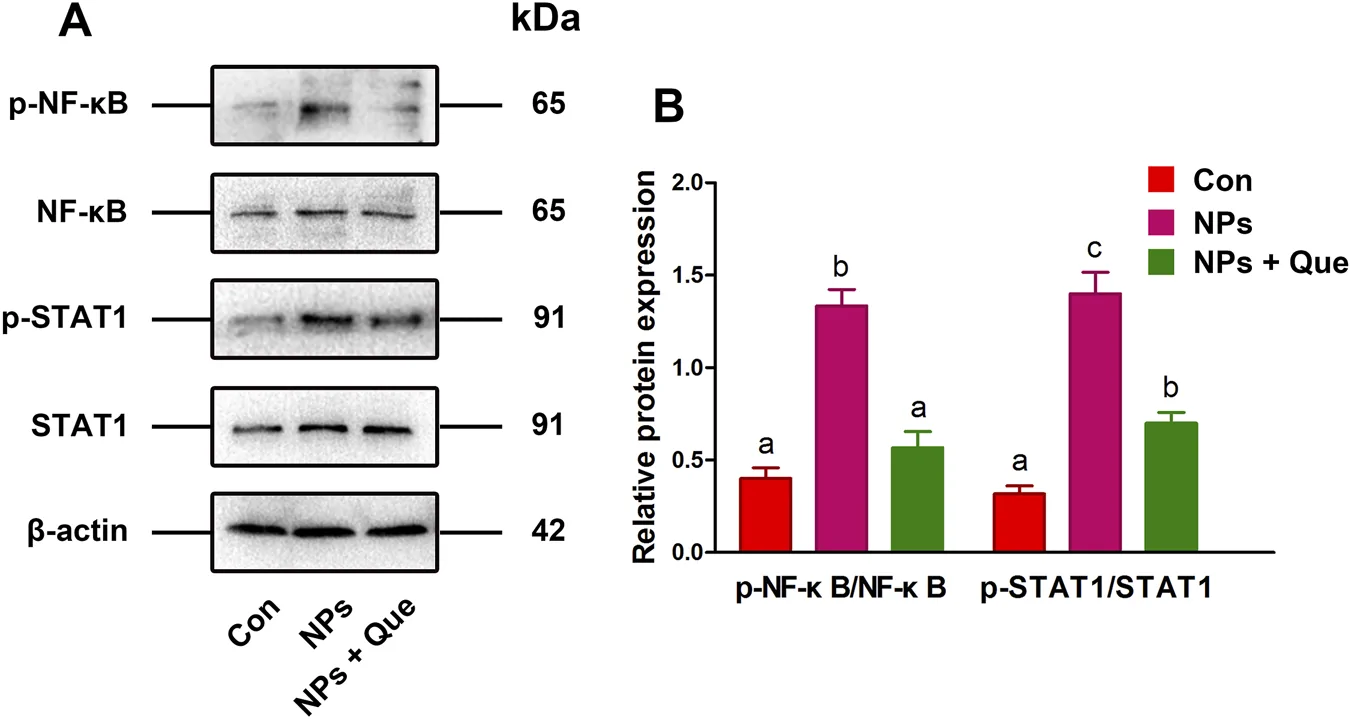
Effects of quercetin on PS-NP-induced activation of NF-κB and STAT1 signaling in RAW 264.7 macrophages. **(A)** Changes in the phosphorylation levels of NF-κB p65 and STAT1 in control cells and cells treated with PS-NPs alone or in combination with quercetin. **(B)** Quantitative analysis of the p-NF-κB p65/NF-κB p65 and p-STAT1/STAT1 ratios. β-actin was used as the loading control. Values are presented as mean ± SD from three independent experiments (*n* = 3). Different letters indicate significant differences among groups (*P* < 0.05).

## 4 Discussion

PS-NPs are emerging environmental contaminants whose potential gastrointestinal toxicity has attracted increasing attention (Li et al., 2024). In the present study, 50 nm PS-NPs were used to investigate intestinal barrier-associated injury and macrophage inflammatory responses using both *in vivo* and *in vitro* models. Our findings showed that PS-NPs exposure was associated with intestinal inflammation, tight junction disruption, and a shift toward a pro-inflammatory macrophage phenotype. Moreover, quercetin attenuated these alterations, potentially through the modulation of macrophage inflammatory responses accompanied by altered NF-κB and STAT1 activation.

In the present study, we first demonstrated in an ICR mouse model that oral administration of PS-NPs at 0.5 and 5 mg/kg/day for four consecutive weeks induced marked pathological alterations in the colon, including mucosal edema, epithelial disruption, and inflammatory cell infiltration, with the severity of injury increasing in a dose-dependent manner. Meanwhile, PS-NPs exposure significantly upregulated the mRNA expression of the pro-inflammatory cytokines *IL-1β*, *TNF-α*, and *IL-6*, while reducing the expression of the tight junction proteins ZO-1 and occludin. These findings suggest that PS-NPs impair intestinal barrier-associated integrity through induction of intestinal inflammation and disruption of tight junction-associated proteins. Although tight junction protein alteration is widely considered an important indicator of intestinal barrier impairment, additional functional permeability assays would further strengthen the evaluation of barrier dysfunction in future studies. This finding is consistent with previous studies showing that nanoplastic exposure induces oxidative stress and inflammatory responses, promotes intestinal epithelial apoptosis and tight junction disruption, and ultimately compromises intestinal barrier integrity (Cui et al., 2023; Zhang et al., 2023; Lee et al., 2024). The PS-NP doses used in the present study represent experimental exposure conditions rather than environmentally relevant exposure levels. Likewise, the quercetin dose used in this study is substantially higher than typical dietary intake and should be considered an experimental intervention dose; therefore, the present findings should not be directly extrapolated to realistic environmental exposure or dietary intake scenarios in humans. Because plasma and tissue levels of quercetin and its metabolites were not measured, the internal exposure associated with the administered dose could not be directly determined and warrants further pharmacokinetic investigation. Future studies should further characterize particle properties in the actual exposure media, including hydrodynamic size, polydispersity index, zeta potential, and endotoxin contamination.

Further investigation revealed that PS-NPs exposure shifted macrophages in mouse colonic tissues toward a pro-inflammatory M1 like phenotype, as evidenced by increased expression of the M1-associated marker CD86 and decreased expression of the M2 associated marker CD206. These findings suggest that dysregulated macrophage inflammatory responses may represent an important link between PS-NP-induced intestinal inflammation and epithelial barrier impairment. However, whether macrophage polarization is a necessary initiating event or a secondary response during PS-NP-induced intestinal injury remains unclear. Future studies using macrophage depletion, selective inhibition of inflammatory macrophage responses, or cytokine neutralization strategies are needed to further establish causal relationships. In the RAW 264.7 macrophage model, PS-NPs directly promoted an M1 like phenotype and upregulated the expression of pro-inflammatory cytokines, corroborating the *in vivo* findings. Previous studies have likewise shown that polystyrene nanoparticles can alter macrophage polarization profiles and that pro-inflammatory M1 macrophages contribute to intestinal epithelial barrier dysfunction (Fuchs et al., 2016; Li et al., 2024). Furthermore, using a Transwell co-culture system, we demonstrated that PS-NP-stimulated macrophages impaired epithelial tight junction-associated protein expression, as indicated by reduced ZO-1 and occludin expression in MODE-K cells. This finding further supports a critical mediating role for macrophage polarization in PS-NP-induced intestinal toxicity.

Quercetin, a naturally occurring flavonoid, has been widely recognized for its anti-inflammatory and immunomodulatory properties (Overman et al., 2011). In the present study, quercetin treatment markedly alleviated PS-NP-induced colonic histopathological alterations, reduced pro-inflammatory cytokine expression, and partially recovered the levels of the tight junction-associated proteins ZO-1 and occludin, suggesting improved intestinal barrier-associated integrity. The restoration of tight junction-associated protein expression may reflect the ability of quercetin to promote epithelial junctional homeostasis, as previously reported (Suzuki and Hara, 2009). Previous studies have further demonstrated that quercetin can protect intestinal epithelial integrity by reducing inflammatory responses and regulating epithelial barrier-related pathways (Overman et al., 2011). Meanwhile, quercetin modulated PS-NP-associated macrophage inflammatory responses, as indicated by decreased CD86 expression and increased CD206 expression. This observation is consistent with previous studies showing that quercetin can regulate macrophage activation states and attenuate excessive inflammatory responses through modulation of inflammatory signaling pathways (Kim and Park, 2016; Tsai et al., 2021). In addition, quercetin has been reported to suppress inflammatory mediator production in RAW 264.7 macrophages through regulation of inflammatory signaling pathways, including NF-κB-related responses (Cho et al., 2003). These findings suggest that macrophage inflammatory modulation may represent an important component of the protective effects of quercetin against PS-NP-induced intestinal injury. Further investigations focusing on macrophage-specific responses will help clarify the precise contribution of macrophage regulation to quercetin-mediated intestinal protection. The co-culture experiments further supported the involvement of macrophage-derived soluble factor-mediated inflammatory communication in regulating intestinal epithelial responses. However, this indirect Transwell model mainly reflects soluble factor-mediated macrophage–epithelial communication and does not fully reproduce the complex intestinal microenvironment involving direct cell–cell interactions, extracellular matrix components, and multiple immune cell populations.

To further explore the inflammatory signaling events associated with quercetin-mediated regulation of macrophage responses, we examined the activation status of the NF-κB and STAT1 signaling pathways. In the present study, PS-NPs exposure significantly increased the phosphorylation levels of NF-κB p65 and STAT1, whereas quercetin treatment reduced these phosphorylation events. NF-κB and STAT1 are recognized as important regulators involved in macrophage inflammatory activation and the expression of inflammatory mediators (Molawi and Sieweke, 2013; Mussbacher et al., 2023). Consistent with our findings, previous studies have reported that polystyrene nanoparticles can activate inflammatory signaling pathways, including ROS-dependent MAPK/NF-κB signaling, thereby promoting inflammatory mediator production in macrophages (Chen et al., 2023). Conversely, inhibition of STAT1 and NF-κB signaling has been implicated in the ability of quercetin to restrain pro-inflammatory macrophage polarization (Fan et al., 2019; Zhou et al., 2023). Therefore, the reduced phosphorylation of NF-κB p65 and STAT1 observed in the present study suggests that altered inflammatory signaling may contribute to the modulatory effects of quercetin on PS-NP-induced macrophage responses. However, because the current study focused on signaling activation patterns rather than pathway-specific intervention, the precise contribution of NF-κB and STAT1 to macrophage polarization requires further investigation.

The use of male mice should be considered when interpreting the present findings, as sex-dependent differences in biological responses to PS-NPs have been reported (Li et al., 2026). Therefore, the current findings should primarily be interpreted within the context of male mice, and future studies including both sexes will be necessary to determine whether the observed intestinal and macrophage responses, as well as the protective effects of quercetin, are sex-dependent. In addition, the sample size was selected based on previous comparable experimental studies and preliminary observations rather than an *a priori* power calculation; therefore, future studies with prospectively determined sample sizes will be valuable for further validating the present findings. Beyond the inflammatory mechanisms examined in the present study, quantitative assessment of PS-NP accumulation represents an important direction for future research. The administered exposure dose does not necessarily reflect the actual internal dose because the uptake, distribution, and retention of nanoplastics may vary among tissues and cell populations. Recent advances in traceable nanoplastic systems, including metal-doped nanoplastics and other quantitative tracing approaches, provide promising tools for sensitive determination of nanoplastic uptake and tissue distribution. In particular, advanced bioimaging approaches based on aggregation-induced emission (AIE) offer the potential for sensitive visualization and spatial localization of nanoplastics in complex biological systems. Such approaches may help resolve the tissue and cellular distribution of nanoplastics and, when integrated with toxicological endpoints, could strengthen the connection between particle biodistribution and biological effects, thereby advancing mechanistic assessment in nanotoxicology (Yan et al., 2025; Li et al., 2026; Yan and Jiang, 2026). Incorporating such approaches in future studies may enable more accurate assessment of intestinal PS-NP burden and cellular accumulation, thereby helping to establish clearer relationships among administered exposure, internal dose, and subsequent inflammatory responses (Qian et al., 2025).

Taken together, our findings support a model in which 50 nm PS-NPs induce intestinal inflammatory responses accompanied by macrophage inflammatory activation and altered NF-κB/STAT1 signaling. The Transwell co-culture results further suggest that macrophage-mediated inflammatory communication contributes to epithelial tight junction-associated alterations. Quercetin attenuated PS-NP-induced intestinal injury, potentially through modulation of macrophage inflammatory responses accompanied by reduced activation of NF-κB and STAT1 signaling (Figure 7). This study provides further insight into the involvement of immune-mediated processes in nanoplastic-induced intestinal toxicity and highlights the potential of natural compounds as modulators of inflammation-associated intestinal injury caused by micro- and nanoplastic exposure.

**FIGURE 7:**
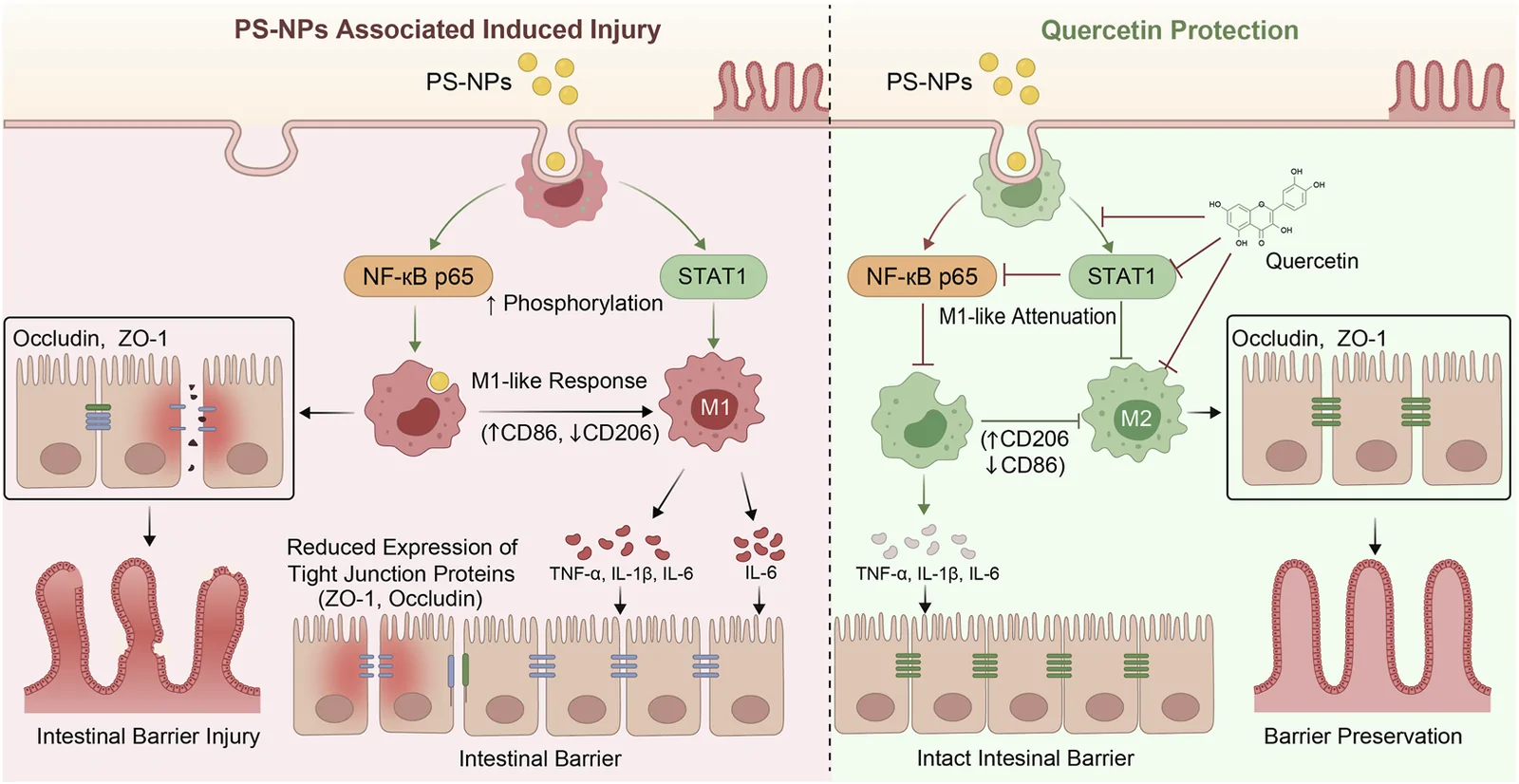
Mechanism diagram of quercetin improving intestinal barrier damage induced by PS NPs. PS-NP exposure was associated with increased NF-κB p65 and STAT1 phosphorylation in macrophages, accompanied by pro-inflammatory macrophage responses characterized by increased CD86 expression and decreased CD206 expression. These inflammatory responses were accompanied by increased secretion of pro-inflammatory cytokines, including TNF-α, IL-1β, and IL-6, which may contribute to reduced expression of tight junction-associated proteins (Occludin and ZO-1) and intestinal barrier injury. Quercetin intervention attenuated these PS-NP-associated inflammatory alterations, as indicated by reduced NF-κB p65 and STAT1 phosphorylation and modulation of macrophage inflammatory responses (decreased CD86 expression and increased CD206 expression). These changes were accompanied by reduced pro-inflammatory cytokine production and preservation of tight junction-associated protein expression, thereby contributing to the maintenance of intestinal barrier integrity.

## Data Availability

The original contributions presented in the study are included in the article/Supplementary Material, further inquiries can be directed to the corresponding author.
