# Supplementary material for: Quercetin attenuates polystyrene nanoplastic-induced intestinal barrier injury through modulation of macrophage inflammatory responses
Source: Front Pharmacol. 2026 Oct 12;17:1973458. doi: 10.3389/fphar.2026.1973458 (PMC13696278; doi:10.3389/fphar.2026.1973458)
Supplement: Supplementary file 1 [file Image1.pdf]

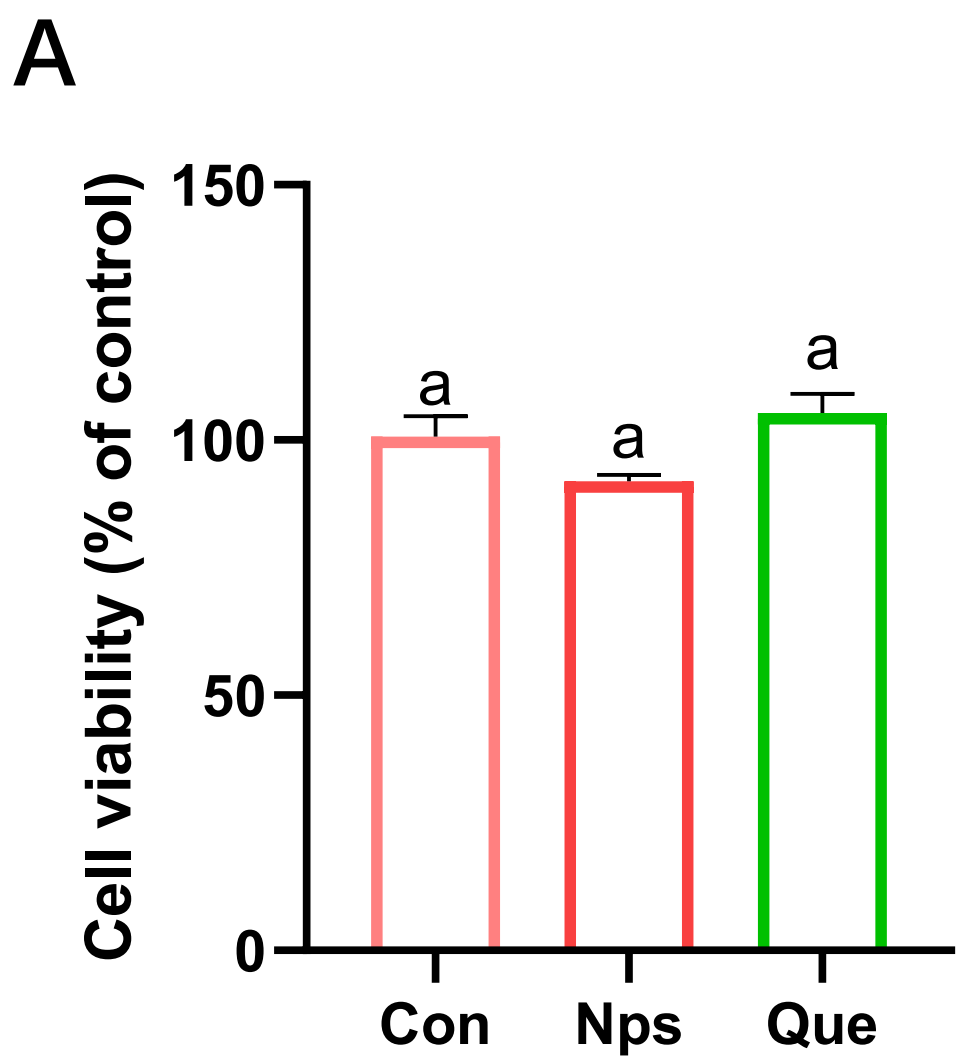

A
B
Cell viability (% of control)
150
100
50
0
a
Con
Nps
Que
